# Development and Pilot Testing of a Questionnaire on Public Attitudes Toward Physician-Assisted Dying, Euthanasia, Advance Directives and End-Of-Life Care

**DOI:** 10.1192/j.eurpsy.2026.11258

**Published:** 2026-07-31

**Authors:** S. Georgakis, E. Dragioti, F. Veroniki, K. Stamatis, G. Papathanakos, M. Gouva, V. Koulouras

**Affiliations:** 1 Intensive Care Unit, University General Hospital of Ioannina; 2 Scientific Laboratory of Psychology & Person-Centered Care, Department of Nursing, University of Ioannina, Ioannina, Greece

## Abstract

**Introduction:**

Concepts such as euthanasia, physician-assisted dying (PAD), advance directives (ADs) and end-of-life (EOL) care remain highly debated yet underexplored at the public level. With existing tools being fragmentary and legal frameworks in many countries still underdeveloped, these gaps underscore the need for a clear and cohesive exploration of attitudes across the social spectrum.

**Objectives:**

This study aims to design and pilot test a comprehensive questionnaire for assessing public perceptions on euthanasia, PAD, ADs and EOL care, integrating personal, legal, ethical, and spiritual dimensions.

**Methods:**

Instrument development involved a literature review, comparative analysis of existing tools and content validation by an expert panel of intensivists, psychologists and legal scholars. Cognitive debriefing was conducted with 15 individuals (professionals and laypersons) to refine clarity and content validity. The revised 50-item instrument, including 5-point Likert scales and sociodemographic items, was pilot tested with 150 Greek adults (Sept–Oct 2024). Internal consistency was assessed via Cronbach’s α and construct validity was explored using principal component analysis (PCA).

**Results:**

Pilot testing supported a clear three-factor structure: Perspectives on Euthanasia and PAD, Perspectives on ADs, and Perspectives on EOL Practices and Palliative Care. The scale demonstrated excellent reliability (total α = 0.913). Minor wording and definitional refinements improved clarity without altering core content.

**Conclusions:**

This study presents the development of a novel instrument for assessing public attitudes toward euthanasia, PAD, ADs, and EOL care, pilot tested in Greece. The results confirm strong psychometric properties and a coherent conceptual structure. A nationwide validation study (N = 1,046) is underway to establish the tool’s reliability and cross-cultural relevance. Beyond academic contribution, the instrument holds potential to inform clinical practice, public policy and ethical debate, offering a robust foundation for official dialogue on such contested and sensitive topics.

**Disclosure of Interest:**

None Declared

